# Valorization of the Invasive Blue Crabs (*Callinectes sapidus*) in the Mediterranean: Nutritional Value, Bioactive Compounds and Sustainable By-Products Utilization

**DOI:** 10.3390/md22090430

**Published:** 2024-09-23

**Authors:** Rosaria Arena, Giuseppe Renda, Giovanna Ottaviani Aalmo, Frédéric Debeaufort, Concetta Maria Messina, Andrea Santulli

**Affiliations:** 1 Laboratory of Marine Biochemistry and Ecotoxicology, Department of Earth and Marine Sciences DiSTeM, University of Palermo, Via Barlotta 4, 91100 Trapani, Italy; rosaria.arena@unipa.it (R.A.); pepperenda76@gmail.com (G.R.); andrea.santulli@unipa.it (A.S.); 2 Department of Economics and Society, Norwegian Institute of Bioeconomy Research (NIBIO), NO-1433 Ås, Norway; giovanna.ottaviani.aalmo@nibio.no; 3 IUT-Dijon-Auxerre, Department of BioEngineering, University of Burgundy, 21078 Dijon Cedex, France; frederic.debeaufort@u-bourgogne.fr; 4 Joint Unit A02.102 PAM-PAPC—Physical Chemistry of Food and Wine Laboratory, University of Burgundy Franche-Comté/AgroSupDijon, 21000 Dijon Cedex, France

**Keywords:** *Callinectes sapidus*, blue crabs, nutrition value, by-products

## Abstract

The blue crab (*Callinectes sapidus*), originally from the western Atlantic Ocean, has recently spread to the Mediterranean and is now considered one of the one hundred most invasive species in that region. This opportunistic species, known for its adaptability to different temperatures and salinities, negatively impacts biodiversity and human activities such as fishing and tourism in the Mediterranean. However, the blue crab is gaining interest as a potential food resource due to its high nutritional value and delicate, sweet flavor. Its meat is rich in protein (14% to 30%), omega-3 fatty acids (EPA and DHA) and other essential nutrients beneficial for human health such as vitamins, and minerals. Utilizing this species in the production of new foods could help mitigate the negative impact of its invasiveness and offer economic opportunities. One challenge with this potential resource is the generation of waste. Approximately 6–8 million tonnes of crab shells are produced worldwide each year, leading to disposal problems and concerns regarding environmental sustainability. To improve economic and environmental sustainability, there is a need to valorize these residues, which are an important source of proteins, lipids, chitin, minerals, and pigments that can be processed into high-value-added products. However, especially in areas with industrial pollution, attention should be paid to the heavy metal (Cd and As) contents of blue crab shells. Studies suggest that blue crab by-products can be used in various sectors, reducing environmental impacts, promoting a circular economy, and creating new industrial opportunities.

## 1. Introduction

The blue crab (*Callinectes sapidus* Rathbun, 1896 (Brachyura: Portunidae)), native to the western Atlantic Ocean [1], is an opportunistic euryhaline species whose life cycle occurs in both brackish and marine habitats, including estuaries, lagoons, and other coastal habitats [2,3].

Blue crabs spend most of their life cycle in estuarine habitats and their distribution depends on temperature, salinity and dissolved oxygen [1]. Temperature plays a key role in the life cycle of this species. Juvenile and adult blue crabs have a tolerance range of 0–39 °C, which may depend on several factors, including the ability of the blue crab to acclimate to both temperature and salinity [1,4]. During the warm season, egg production and incubation occur, while feeding, movement, and moulting cease when temperatures drop below 10 °C [5]. In terms of salinity, blue crabs use various salinity regimes to complete their life cycle. For example, they use low-salinity environments for mating and as nursery habitats, and seawater salinity levels for larval growth [6]. Therefore, blue crabs can tolerate and grow in a wide range of environmental salinities [1]. Furthermore, blue crabs disperse along a salinity gradient according to sex and life stage: males and juveniles prefer oligohaline waters upstream, while adult females select areas with meso-and polyhaline salinities [7]. With regard to the parameter of dissolved oxygen (DO), it was demonstrated that this has a significant effect on the growth and abundance of blue crabs. Levels of DO below 2–4 mg/L were shown to induce an avoidance response in crabs, while exposure to levels below 1.2 mg/L was observed to result in mortality [7].

The successful spread of blue crabs across regions is due to their biological characteristics: rapid growth rates, opportunistic feeding (on vegetation, detritus, polychaetes, molluscs, crustaceans and fish), high reproductive rate, generalist habitat uses, long-distance larvae dispersal, and effective physical and aggressive behaviours, including scavenging and cannibalistic [1,8,9,10,11]. Due to these characteristics, the blue crab, accidentally introduced in the early 20th century in the Mediterranean Sea, likely through ballast water [12] and deliberately introduced in the Aegean Sea in the 1930s [13], has since spread widely in the Mediterranean, the Black Sea, and along the European coasts of the Atlantic Ocean [2]. Thus far, blue crabs are considered one of the hundred “worst invasive” alien marine species in the Mediterranean Sea, with a high impact on ecosystem biodiversity and human activities (fisheries, aquaculture, tourism) [14,15]. This species has the potential to cause radical changes in the composition and structure of marine communities in coastal zones by competing with native species for resources [1,16]. It has a severe impact on artisanal fisheries, reducing both the quantity and quality of catches, and negatively affecting overall income [9]. Despite the negative impacts, the blue crab could be considered a new source of income since, considering its high nutritional value [8,9,15,17], its harvesting could improve the economic situation of both the local fishing [8,15] and processing sectors through its utilization in local industries and the valorization of by-products [8,15].

## 2. Commercial Value and Management Approach

The blue crab holds substantial commercial value, particularly in its native range along the western Atlantic Ocean and the Gulf of Mexico. In these regions, it is a cornerstone of the fishing industry, contributing significantly to local economies through job creation in harvesting, processing, distribution and sales sectors [18].

In the Mediterranean Sea, the blue crab has become an invasive species, posing significant ecological and economic challenges, particularly in regions such as Sicily [9,19].

The blue crab represents a double-edged sword for local ecosystems and economies. Ecologically, it can alter local marine life by preying on native species and competing for resources, reducing biodiversity and affecting fisheries. In addition, blue crab management is costly, as traditional disposal methods are expensive and laborious, with a low yield of usable meat.

On the other hand, turning the invasive blue crab into a commercial resource offers a novel solution in the European context to mitigate its negative impacts. By developing products such as crab patties, pasta sauces, and crab burgers, businesses can create economic value from what is otherwise considered a nuisance. This approach aligns with broader strategies seen in other regions, where invasive species are harvested for food, pharmaceuticals, or cosmetic products, thereby controlling their populations while generating revenue. This approach was successfully implemented in Sicily where a seafood enterprise has integrated innovative practices to manage and utilize the blue crab, developing a range of blue crab-based products. These efforts have focused on creating value-added products tailored to both local and national markets, despite the inherent challenges associated with the species’ low meat yield and high processing costs.

The effective commercialization of blue crab products necessitates robust infrastructure and advanced technological applications. Significant investments have been made in novel preservation methods, such as modified atmosphere packaging, to extend the shelf life of blue crab products. Additionally, the implementation of mechanized processing systems is emphasized to reduce labour costs and enhance operational efficiency.

While the current infrastructure adequately supports existing operations, several challenges persist, including the need for more efficient processing technologies and improved integration within supply chain and logistics frameworks. Future plans are directed towards upgrading facilities to enhance both production capacity and operational efficiency, reflecting a broader industry trend towards modernization and technological integration within the seafood sector.

Regulatory frameworks play a critical role in shaping the economic viability of utilizing blue crabs. Until recently, the use of specific fishing gear and the classification of blue crabs as a marketable species were major regulatory hurdles. For instance, the Veneto Region received a special allowance to use dredges within three miles of the coast to prevent damage to mussel beds. These regulatory adjustments are crucial for enabling businesses to legally and efficiently harvest blue crabs.

Market perception and consumer acceptance are also vital. In Sicily, the blue crab is not yet seen as a significant threat, but its potential to become one is recognized. Developing a market for blue crab products involves not only creating appealing products but also educating consumers about the benefits of using this invasive species as a sustainable food source.

## 3. Nutritional Aspects of *C. sapidus*

In the Mediterranean, there is a growing interest in the potential of the blue crab as a food resource due to its high nutritional value [8,9,15,17]. The meat, characterized by a delicate and sweet flavour and sold at a high market price (averaging USD 35.84 per pound [20]), is rich in essential nutrients such as omega-3 fatty acids, vitamins, and minerals. These nutritional components, beneficial for human health, combined with the low fat content, contribute to improving cardiovascular performances in terms of heart health, brain function, and overall well-being [21].

The proximate composition in terms of moisture, lipid, protein, and ash, as well as the main fatty acids reported by different studies on the edible meat of the blue crab, are summarized in Table 1. Differences in biochemical composition among individuals of the same species can be attributed to various factors, such as habitat influence, which is related to different diets with different biochemical compositions that directly affect the crab, exposure to different environmental conditions, and resource availability [21]. Seasonal and climatic changes also play a fundamental role in shaping the biochemical composition of this species. Environmental factors such as temperature, salinity, light, and nutrient availability throughout the year can impact their metabolism and biochemical processes, leading to variations in their composition. Biological differences, such as size, age, sex, maturity stage, gametogenesis, and spawning cycle, can also influence the biochemical composition of the blue crab [20,21,22,23,24].

In terms of nutritional composition, blue crab meat is rich in protein, typically ranging from 14% to 30%. This protein content is influenced by seasonal variations, with the lowest levels observed in autumn for both males and females and the highest values in the spring season, which could be due to the change in the carapace of blue crabs in autumn [25]. On this basis, the consumption of 50 g of *C. sapidus* meat can meet the World Health Organisation’s (WHO) recommended daily requirement of essential amino acids for an adult [26,27].

*C. sapidus*, like other crustaceans, has a low lipid content, typically between 0.4% and 4% (Table 1), making it suitable for low-fat diets [15,26,28,29]. The difference in lipid content between males and females is consistent across all seasons, with females consistently having significantly higher lipid content than males. Furthermore, both male and female crabs have higher lipid content in winter [25,30]. This is probably due to a metabolic strategy used by crustaceans to store high-energy compounds that will be useful during winter [30,31]. Furthermore, a markedly elevated fat content was observed in leg meat relative to claw meat [25,32]. This phenomenon can be attributed to a number of factors influencing the lipid content in seafood, including environmental conditions, seasonality, sex and sexual variations related to reproduction, and the carapace moulting period [25].

Regarding fatty acids profile, the predominant class of fatty acids is polyunsaturated fatty acid (PUFA) in all seasons. These include arachidonic acid (ARA; C20:4 n6), eicosapentaenoic acid (EPA; 20:5 n-3) and docosahexaenoic acid (DHA 22:6 n-3) [15,25,29,30,32].

Several studies have shown that blue crab, despite its low fat content, is an excellent source of n-3 PUFA, particularly EPA and DHA, throughout the year, for both sexes and in all muscle types. Its consumption could meet the recommended dietary requirement of n-3 fatty acids (DHA and EPA) in the human diet [15,22,25,28,29,30,32].

Despite the high nutritional value of blue crab meat, the marketing and processing of this species are closely linked to meat yield, which is very low for this species. Meat yield depends on several factors, such as sex and processing methods. In fact, male crabs generally have a higher yield than females, as the latter consume more energy for vitellogenesis during the reproductive period. The yield also varies depending on whether the blue crabs are fresh or cooked and whether the product is processed mechanically or manually [15,20,33,34]. It was observed that using mechanical processing of the blue crab and rock crab by-products, after hand picking, can increase the amount of meat available for the production of new food products by up to 20% [35,36,37,38,39].

The potential applications of crab meat in producing crab-based products have been extensively studied in various crab species [21]. These studies have shown promising results, indicating that the same approach can be applied to the blue crab [21]. By recognizing the blue crab as a new food source and exploring its potential applications, we can effectively tackle the challenges posed by its invasive nature [40]. Moreover, this approach opens up exciting economic opportunities and promotes sustainable practices in the Mediterranean region. By utilizing the blue crab in the production of crab-based products, it is possible not only to reduce the negative impact of its invasive presence but also to contribute to the local economy and foster environmentally friendly practices.

**Table 1:** Proximate and fatty acids composition of edible blue crabs.

	Moisture (%)	Lipid (%)	Protein (%)	Ash (%)	Fatty Acids %	Season	Geographic Area	**References**
Crab meat	78.71		18.40	2.32	-	-	local market (Madrid, Spain)	[20]
Crab meat (cooked)	80.17		16.46	2.32	-	-		
Claw meat	83.1	0.64	15.0	1.39	-	Summer	Gulf of Antalya, the coast of Western Mediterranean of Turkey	[28]
Body meat	81.58	0.79	14.71	1.89	-	Summer		
Claw meat (cooked)	78.02	0.44	19.55	2.13	-	Winter	Akyazan lagoon on the coast of the eastern Mediterra- nean Sea of Turkey	[26]
Breast meat (cooked)	79.05	0.44	18.10	2.03	-	Winter		
Claw meat	80.12	-	15.13	1.63	-	Summer	Acquatina Lagoon (SE Italy)	[41]
Breast meat (cooked)	-	-	-	-	n3PUFA = 17.0; ARA = 5.02; EPA = 8.4; DHA = 6.8,	Winter	Off the Mediterranean coast of Akyazan Lagoon Karataş/Turkey	[29]
Claw meat (cooked)	-	-	-	-	n3PUFA = 18.1; ARA = 7.48; EPA = 10.6; DHA = 5.9,	Winter		
Lump crab meat (female)	74.33	1.24	21.64	2.18	SFA = 25.24; MUFA = 26.24; PUFA = 38.94; ARA = 4.93; EPA = 17.89; DHA = 12.02	Spring	Eastern Mediterranean Sea	[25]
Claw crab meat (female)	72.48	0.80	24.33	2.24	SFA = 23.65; MUFA = 28.04; PUFA = 37.88; ARA = 6.38; EPA = 16.89; DHA = 10.34	Spring	Eastern Mediterranean Sea	
Lump crab meat (male)	74.98	1.22	21.41	2.25	SFA = 24.74; MUFA = 23.22; PUFA = 42.64; ARA = 5.84; EPA = 17.91; DHA = 14.13	Spring	Eastern Mediterranean Sea	
Claw crab meat (male)	74.65	0.92	22.25	2.07	SFA = 23,00; MUFA = 25.86; PUFA = 41.13; ARA = 7.68; EPA = 16.95; DHA = 12.22	Spring	Eastern Mediterranean Sea	
Lump crab meat (female)	76.01	0.86	20.79	2.09	SFA = 28.25; MUFA = 25.39; PUFA = 39.25; ARA = 5.29; EPA = 17.48; DHA = 13.07	Summer	Eastern Mediterranean Sea	
Claw crab meat (female)	73.36	0.81	23.32	2.35	SFA = 27.02; MUFA = 27.33; PUFA = 39.25; ARA = 6.11; EPA = 17.83; DHA = 11.55	Summer	Eastern Mediterranean Sea	[25]
Lump crab meat (male)	77.36	1.01	19.19	2.33	SFA = 28.63; MUFA = 22.64; PUFA = 41.85; ARA = 6.07; EPA = 16.32; DHA = 15.40	Summer	Eastern Mediterranean Sea	
Claw crab meat (male)	76.46	0.69	20.25	2.48	SFA = 27.38; MUFA = 25.75; PUFA = 40.10; ARA = 7.29; EPA = 15.92; DHA = 12.81	Summer	Eastern Mediterranean Sea	
Lump crab meat (female)	80.83	0.89	16.61	1.53	SFA = 27.41; MUFA = 27.48; PUFA = 39.77; ARA = 6.10; EPA = 19.19; DHA = 10.64	Autumn	Eastern Mediterranean Sea	
Claw crab meat (female)	76.73	0.63	20.17	2.06	SFA = 28.00; MUFA = 31.08; PUFA = 34.22; ARA = 6.66; EPA = 15.05; DHA = 8.70	Autumn	Eastern Mediterranean Sea	
Lump crab meat (male)	77.64	0.98	19.48	1.72	SFA = 26.04; MUFA = 25.04; PUFA = 41.76; ARA = 5.79; EPA = 19.75; DHA = 11.72	Autumn	Eastern Mediterranean Sea	
Claw crab meat (male)	79.64	0.58	17	2.13	SFA = 25.85; MUFA = 30.46; PUFA = 37.06; ARA = 6.65; EPA = 16.75; DHA = 9.02	Autumn	Eastern Mediterranean Sea	
Lump crab meat (female)	76.45	1.21	20.48	1.76	SFA = 21.63; MUFA = 22.89; PUFA = 41.60; ARA = 8.96; EPA = 18.50; DHA = 9.83	Winter	Eastern Mediterranean Sea	
Claw crab meat (female)	75.06	0.84	21.80	2.08	SFA = 22.09; MUFA = 23.11; PUFA = 39.60; ARA = 9.98; EPA = 16.91; DHA = 8.60	Winter	Eastern Mediterranean Sea	
Lump crab meat (male)	77.09	1.51	19.20	1.83	SFA = 24.82; MUFA = 23.84; PUFA = 44.01; ARA = 7.45; EPA = 19.25; DHA = 13.02	Winter	Eastern Mediterranean Sea	
Claw crab meat (male)	75.66	1.04	21.05	2.06	SFA = 23.64; MUFA = 27.81; PUFA = 40.14; ARA = 9.24; EPA = 17.56; DHA = 9.35	Winter	Eastern Mediterranean Sea	[25]
Cephalothorax muscle (female)	68.8	2	26.56	-	SFA = 30.31; MUFA = 26.06; PUFA = 43.64; ARA = 7.18; EPA = 15.64; DHA = 13.27	Summer	Southern lagoon of Tunis (north-east of Tunisia) during	[15]
Chelipeds muscle (female)	70.3	3.69	23.71	-	SFA = 31.64; MUFA = 26.35; PUFA = 42.01; ARA = 7.43; EPA = 13.83; DHA = 13.78	Summer	Southern lagoon of Tunis (north-east of Tunisia) during	
Cephalothorax muscle (male)	67.4	2.91	27.05	-	SFA = 32.04; MUFA = 23.80; PUFA = 44.15; ARA = 7.21; EPA = 15.20; DHA = 13.29	Summer	Southern lagoon of Tunis (north-east of Tunisia) during	
Chelipeds muscle (male)	68.7	2.86	25.77	-	SFA = 31.74; MUFA = 25.59; PUFA = 42.67; ARA = 9.21; EPA = 12.58; DHA = 11.25	Summer	Southern lagoon of Tunis (north-east of Tunisia) during	
Body meat (female)	67.54	1.62	26.51	1.16	SFA = 25.98; MUFA = 22.04; PUFA = 41.41; ARA 8.71; EPA = 15.20; DHA = 11.43	-	Akyatan Lagoon in the south of the Mediterranean Sea	[32]
Claw meat (female)	68.12	1.12	29.59	1.58	SFA = 24.75; MUFA = 23.57; PUFA = 41.60; ARA = 10.89; EPA = 13.11; DHA = 11.23	-	Akyatan Lagoon in the south of the Mediterranean Sea	
Body meat (male)	66.40	1.64	30.31	1.10	SFA = 26.76; MUFA = 21.79; PUFA = 39.73; ARA = 7.73; EPA = 13.61; DHA = 13.03	-	Akyatan Lagoon in the south of the Mediterranean Sea	
Claw meat (male)	66.59	1.22	31.03	1.32	SFA = 24.62; MUFA = 23.84; PUFA = 40.98; ARA = 10.59; EPA = 13.0; DHA = 10.84	-	Akyatan Lagoon in the south of the Mediterranean Sea	
Body meat (female)	67.99	2.02	27.69	1.15	SFA = 31.84; MUFA = 7.55; PUFA = 43.78; ARA = 9.49; EPA = 16.29; DHA = 9.45	-	Hurma Strait in the south of the Mediterranean Sea	
Claw meat (female)	68.15	1.39	26.78	1.64	SFA = 24.13; MUFA = 23.26; PUFA = 41.38; ARA = 10.9; EPA = 13.01; DHA = 9.51	-	Hurma Strait in the south of the Mediterranean Sea	[32]
Crab meat	83.59	1.80	9.12	1.22	SFA = 2733.67 mg/100 g of sample; UFA = 1061.93 mg/100 g of sample	Summer	Baltim Fish Market, Kafar Elshaikh, Egypt during	[42]
Carapace meat (male)	77.69	0.69	18.97	2.39	-	-	Fatsa, Ordu, Türkiye	[22]
Left Claw meat (male)	78.32	0.59	18.69	2.38	-	-	Fatsa, Ordu, Türkiye	
Right Claw meat (male)	78.58	0.58	18.26	2.35	-	-	Fatsa, Ordu, Türkiye	
Legs meat (male)	77.49	0.46	19.22	2.34	-	-	Fatsa, Ordu, Türkiye	
Carapace meat (female)	76.73	0.92	19.93	2.29	-	-	Fatsa, Ordu, Türkiye	
Left Claw meat (female)	77.85	0.79	18.75	2.32	-	-	Fatsa, Ordu, Türkiye	
Right Claw meat (female)	78.62	0.77	18.05	2.31	-	-	Fatsa, Ordu, Türkiye	
Legs meat (female)	76.98	0.63	19.72	2.30	-	-	Fatsa, Ordu, Türkiye	
Claw and body meat (female)		0.9	-	-	SFA = 29.0; MUFA = 21.7; PUFA = 39.5; ARA = 6.8; EPA = 15.0; DHA = 12.2	Autumn	Koycegiz Lagoon (Mugla, Turkey)	[30]
Claw and body meat (female)	-	1.3	-	-	SFA = 26.9; MUFA = 22.6; PUFA = 39.5; ARA = 8.1; EPA = 14.1; DHA = 12.3	Winter	Koycegiz Lagoon (Mugla, Turkey)	
Claw and body meat (female)	-	0.9	-	-	SFA = 26.2; MUFA = 21.2; PUFA = 41.1; ARA = 6.7; EPA = 15.0; DHA = 13.8	Spring	Koycegiz Lagoon (Mugla, Turkey)	
Claw and body meat (female)	-	0.9	-	-	SFA = 30.2; MUFA = 21.7; PUFA = 40.4; ARA = 7.9; EPA = 16.3; DHA = 11.1	Summer	Koycegiz Lagoon (Mugla, Turkey)	[30]
Claw and body meat (male)	-	0.8	-	-	SFA = 28.01; MUFA = 21.4; PUFA = 41.7; ARA= 8.0; EPA = 14.5; DHA = 13	Autumn	Koycegiz Lagoon (Mugla, Turkey)	
Claw and body meat (male)	-	1.2	-	-	SFA = 253; MUFA = 22.6; PUFA = 41.6; ARA = 7.7; EPA = 14.0; DHA = 14.9	Winter	Koycegiz Lagoon (Mugla, Turkey)	
Claw and body meat (male)	-	0.8	-	-	SFA = 26.3; MUFA = 18.5; PUFA = 46.5; ARA = 7.4; EPA = 16.9; DHA = 15.9	Spring	Koycegiz Lagoon (Mugla, Turkey)	
Claw and body meat (male)	-	0.8	-	-	SFA = 29.23; MUFA = 18.9; PUFA = 43.1; ARA = 8.5; EPA = 16.0; DHA = 13.0	Summer	Koycegiz Lagoon (Mugla, Turkey)	
Crab muscle (female)	75.14	3.75	9.38	-	-	-	El-Gamil is an outlet located along the northeast side of Lake Manzala, within the coastal sand bar 5 km west of Port Said on the Mediterranean Sea	[43]
Crab muscle (male)	74.38	4.77	11.51	-	-	-	El-Gamil is an outlet located along the northeast side of Lake Manzala, within the coastal sand bar 5 km west of Port Said on the Mediterranean Sea	
Raw crab meat	81.17	1.06	16.22	1.45	-		Köyceğiz Dalyan	[44]
Steam-cooked crab meat	76.88	1.46	18.05	1.88	-		Köyceğiz Dalyan	

ARA: arachidonic acid; DHA: Docosahexaenoic acid; EPA: Eicosapentaenoic acid; MUFA: Monounsaturated fatty acid; PUFA: Polyunsaturated fatty acid; SFA: Saturated fatty acid.

## 4. Blue Crab By-Products Valorization

### 4.1. Shell Valorization

Globally, around 6–8 million tons of crab shells are produced each year, causing disposal problems and environmental concerns [45,46]. Due to its low economic value and high disposal costs, most of this waste is actually disposed of in landfills. To improve the economic and environmental sustainability of crab shell residues, it is important to valorize this biomass and no longer consider it merely waste to be disposed of [45,46,47,48].

Crab shells and other crab by-products are an important source of proteins, lipids, chitin, minerals (mainly calcium and magnesium carbonates) and pigments that can be processed into high-value products [21,45,46,49,50,51,52]. Although the valorization of crustacean by-products has been widely discussed [45,53,54,55,56,57,58,59,60,61,62,63,64,65,66,67,68,69,70,71,72,73,74,75,76,77,78], very few studies have focused specifically on blue crab (*C. sapidus*) [48,51,52,79,80,81,82,83,84,85,86,87,88]. These by-products hold significant potential for the extraction of valuable compounds, including chitin, which is of particular interest for various industrial applications. Table 2 details the studies conducted on blue crab by-products.

The crustacean shells of blue crabs are primarily composed of chitin [53], a linear N-acetyl-d-glucosamine polysaccharide with β-(1-4) linkage, insoluble in water [89], making it the second most abundant biopolymer in the world after cellulose. Chitin is found in the exoskeletons of insects, the cell walls of fungi and algae, and marine invertebrates like crustaceans, including shrimp and crabs [82,83,90,91]. As shown in Table 2, most studies on blue crab (*C. sapidus*) by-products have focused on chitin extraction [51,82,83,84]. Chitin and its derivatives have attracted significant interest in research and industry due to their multiple applications in different sectors (food, agriculture, pharmaceutical and chemical) [77,82,92,93], making it important to identify new resources [79]. Studies on blue crab by-products have shown that they are a good source of chitin [51,79,82,83,84]. Chitin was obtained by treating blue crab shells with alkalis and acids to remove proteins and minerals. This method allowed for obtaining high-quality chitin, with a recovery rate of 10–14% [51,79,82,83,84].

By deacetylating chitin, chitosan is obtained—a polysaccharide with many applications in different fields (clinical, cosmetic, food, pharmaceutical, agricultural, aquaculture and environmental) due to its non-toxicity, biodegradability, and adsorption and chelating ability [57]. Chitosan can also be used as an alternative antimicrobial agent as it possesses antimicrobial activity against many bacteria, fungi and yeasts [79]. Furthermore, chitosan has excellent film-forming properties, making it suitable for applications in the food industry to preserve the quality of various foods [85,94,95,96].

Baron et al. [85], Kaya et al. [79], and Metin et al. [83] obtained chitosan from blue crab by-products with yields ranging from 7 to 14%. These authors showed that chitosan derived from blue crab by-products exhibited antimicrobial activity against human and fish pathogens and was also a good source of antioxidants [79,83]. Consequently, it could be considered a natural alternative to commercial chitosan and synthetic antimicrobials [79,83], with potential application as a biopolymer in the food industry [83]. In addition, chitosan-based biofilms have shown properties that allow their future use as coatings or packaging materials for food products [85].

Blue crab (*C. sapidus*) shells are a valuable source of high-nutritional-value compounds, including proteins, which represent approximately 30% of their content [51,86]. These proteins could serve as excellent precursors for bioactive peptides derived through the protein hydrolysis process [53,72]. Protein hydrolysates and peptides obtained from crustacean by-products have shown significant health benefits, particularly in the prevention of chronic diseases [53,97]. Despite the crucial importance of protein hydrolysates, current literature includes only one study highlighting the possibility of obtaining protein hydrolysates from blue crab by-products [51]. This represents a research and development opportunity to further explore the potential of blue crab by-products not only as a source of high-quality protein but also as nutraceutical ingredients that can significantly contribute to human health.

Other studies have focused on various innovative and sustainable applications of blue crab shells. One such study investigated the production of new carbonaceous materials with high adsorption capacity, showing that carbonaceous materials pyrolyzed at 800 °C have the highest adsorption rate [48]. These carbonaceous materials can be used as effective adsorbents to reduce contaminants in the marine ecosystem and mitigate environmental pollution [48]. Another study showed that the three-dimensional nanostructured natural material derived from blue crabs retains its nanomorphology even after mechanical grinding or heat treatment, making it compatible with a wide range of industrial processes [98]. This result paves the way for numerous innovative applications based on nanoscale porosity and chemical composition of materials after industrial processing steps.

The potential use of blue crab shells as an alternative filler material in the rubber industry was also investigated. The results showed that incorporating these by-products into the ethylene propylene diene monomer (EPDM) matrix improved the rheological, mechanical and thermal properties of the composite. Specifically, the addition of up to 12 phr of BCS to the EPDM matrix was found to increase the T50 temperature, tensile strength and cross-link density of the composite [87]. These biodegradable and bio-based materials may prove to be significant substitutes, offering economic and environmental benefits.

Finally, the biogenic particulate matter derived from blue crab shells was investigated to determine its potential future use in composite formulations for high-value-added products. The analyses showed that these powders meet the legal standards for fertilizers in terms of composition and microbiological content, making them potentially useful as biofertilisers and other sustainable products. However, special attention should be paid to the content of heavy metals in crab shells, especially Cd and As, in areas where industrial pollution is known to occur [88].

**Table 2:** Blue crab by-products, bioactive compounds extracted/isolated and their uses.

Blue Crab By-Products	Bioactive Compounds Extracted or Products	Parameters Evaluated	References
Shell waste	Astaxanthin (extracted with a mixture of supercritical CO, and ethanol as a co-solvent	Chemical composition of demineralized crab shell waste	[86]
Shell	Chitin	Comparison in the chitin extracted from blue edible crab and market purchased chitin.	[82]
Shells and tongs	Chitosan to produce chitosan:pectin (Ch:P) based films	Chitosan production and characterization Film characterizations	[85]
Shell wastes	Chitin and Chitosan	Yield, Moisture and Ash Content of Chitin and Chitosan	[83]
Shells	Chitin and Chitosan	Characterization of chitin and chitosan through FTIR, TGA, XRD, and SEM analyses. Determination of antimicrobial and antioxidant activities of chitosan.	[79]
Shells, pincers, and legs	Produce new carbonaceous materials with high adsorption capacity	The adsorption properties of the materials were investigated	[48]
Shells	Production of Biogenic Micropowders of Crab Shells.	Characterization of Biogenic Micropowders of Crab Shells.	[98]
Shell	Ethylene pro-pylene diene termonomer (EPDM) and EPDM with different blue crab shell (BCS) incorporated into rubber matrix	The effect of carbon black and blue crab shell content within the Ethylene pro-pylene diene termonomer matrix on the rheological, mechani- cal, morphological, and thermal properties	[87]
Shells waste	Plant biofertilizer	Crab Shell Powdering Technical Aspects. Macronutrients, heavy metals, bacterial content, and antimicrobial properties were tested	[88]
Food waste from cooked crabs	Chitin-based polymeric materials	The process of waste shells demineralization and the final products of the treatment were investigated using various Raman techniques and technologies, cross-validating the results with FT-IR, XRD and SEM-EDX techniques.	[84]
Carapaces and legs	Astaxanthin Chitin Hydrolysates protein	Characterization of blue crab processing waste Enzymatic hydrolysis Astaxanthin recovery and antioxidant activity Chitin characterization	[51]
Exoskeleton (carapace and legs)	Astaxanthin	Recovery and quantification of astaxanthin, antioxidant and anti-inflammatory activity antimicrobial activity of extracted astaxanthin	[52]
Exoskeletons	Astaxanthin	Quantification of astaxanthin, extracted with organic solvents (ethyl acetate, acetone, cyclohexane, isopropyl alcohol, hexane, heptane, and a combination of hexane–acetone–ethanol–toluene solvents), by high-performance liquid chromatography analysis.	[80]
Exoskeleton powder	Carotenoids	Composition and morphology of the exoskeleton powder analysed by SEM, Raman spectroscopy, FTIR and XRD.	[81]

### 4.2. Exoskeleton and Cephalothorax Valorization

The exoskeleton and cephalothorax of the blue crab are composed of carotenoids, particularly astaxanthin [51,52,80,81,86]. Astaxanthin is the major carotenoid pigment naturally occurring in marine crustaceans, known for its antioxidant and anti-inflammatory properties [52,99], and for its beneficial effects on human and animal health, which has significantly increased its demand in the market [52,99,100].

Astaxanthin was extracted from blue crab by-products using organic solvent extraction techniques [51,52,80,81] and with supercritical fluids (SFE) using ethanol as co-solvent [86]. As observed by Abd El-Ghany et al. [52], the rigid structure of the blue crab shell, which includes complex proteins and chitin, makes the extraction process difficult. To improve the efficiency of astaxanthin extraction, these authors pre-treated the blue crab by-products with chitinolytic and/or proteolytic microorganisms. Other authors, on the other hand, demineralized the sample before astaxanthin extraction [51,86], thereby increasing the availability of carotenoids and facilitating their extraction with organic solvents [51]. Mixtures of acetone/hexane (1:1 *v*/*v*) [52] and hexane–acetone–ethanol–toluene [80] have shown better efficiency. These studies [51,52,80,81,86] highlighted the potential use of blue crab by-products as a valuable source of carotenoids, particularly astaxanthin. The recovered astaxanthin can be used in various sectors, including food, pharmaceuticals and cosmetics, providing a sustainable and natural alternative to synthetic carotenoids.

These studies demonstrate that blue crab by-products can be employed in various sectors to generate bioactive compounds, thereby reducing environmental impact and advancing a circular economy. Additionally, this approach increases the economic value of marine by-products and creates new avenues for industrial growth.

## 5. The Potential Future of Blue Crab Valorization

Addressing the challenges posed by the blue crab invasion also requires social innovation, which involves new strategies, concepts, and ideas that meet social needs and create new social relationships or collaborations. This can include community-based management programs, educational initiatives, and public–private partnerships. Community-Based Management Programs can engage local communities in the management and utilization of blue crab populations. By involving fishermen, local businesses, and residents in decision-making processes, these programs can ensure that the solutions are locally appropriate and sustainable. For instance, fishermen can be trained to catch blue crabs using methods that minimize ecological damage, while local businesses can be incentivized to develop and market blue crab products. Similarly, educational Initiatives raise awareness about the blue crab issue and its potential solutions can inform the public about the ecological impacts of the blue crab and the benefits of its commercial utilization. Schools, community centre, and media can play pivotal roles in disseminating this information, thereby fostering a culture of sustainability and environmental stewardship. Also, collaboration between government agencies, private enterprises, and academic institutions can drive innovation and investment in blue crab management and utilization. Such partnerships can facilitate research and development, streamline regulatory processes, and provide funding for new initiatives. For example, partnerships can support the development of new processing technologies or the exploration of alternative uses for blue crab by-products. By integrating social innovation into the management of the blue crab invasion, it is possible to create comprehensive and sustainable solutions that not only address the ecological and economic challenges but also enhance community resilience and social cohesion.

## 6. Conclusions

The blue crab (*C. sapidus*) is a significant invasive species in the Mediterranean Sea, presenting both challenges and opportunities. On the one hand, its presence disrupts local ecosystems, leading to negative impacts on biodiversity and human activities such as fishing and tourism. The species competes with native marine life for resources, potentially leading to declines in native populations and altering the ecological balance.

On the other hand, the blue crab’s high nutritional value offers a unique opportunity to turn this invasive species into an economic asset. Its meat is rich in proteins, omega-3 fatty acids, and other essential nutrients, making it a valuable resource for the local fishing and industrial processing sectors. Additionally, the valorization of blue crab by-products—such as proteins, lipids, chitin, minerals, and pigments—holds promise for various industries, including food, pharmaceuticals, and cosmetics. However, care must be taken to avoid the possible presence of heavy metals (Cd and As) in blue crab shells in areas subject to industrial pollution. This approach not only helps mitigate the environmental impact of blue crab invasions but also promotes a circular economy by transforming waste into high-value products.

However, the process of integrating blue crab into local economies is not without its challenges. The low meat yield and high processing costs can make it difficult to commercialize on a large scale. Furthermore, while the potential benefits of utilizing blue crab by-products are significant, more research is needed to fully understand and optimize the bioactivity and industrial applications of these components.

Addressing the blue crab invasion effectively requires a balanced approach that combines economic utilization with ecological management. Social innovation plays a critical role in this effort, with strategies such as community-based management programs, educational initiatives, and public–private partnerships being essential. Engaging local communities, fostering education, and promoting collaboration between various stakeholders can help develop sustainable solutions that not only mitigate the ecological impact of the blue crab but also enhance economic resilience.

## References

[1] Kevrekidis K., Kevrekidis T., Mogias A., Boubonari T., Kantaridou F., Kaisari N., Malea P., Dounas C., Thessalou-Legaki M. (2023). Fisheries Biology and Basic Life-Cycle Characteristics of the Invasive Blue Crab *Callinectes sapidus* Rathbun in the Estuarine Area of the Evros River (Northeast Aegean Sea, Eastern Mediterranean). J. Mar. Sci. Eng..

[2] Mancinelli G., Bardelli R., Zenetos A. (2021). A global occurrence database of the Atlantic blue crab *Callinectes sapidus*. Sci. Data.

[3] Taybi A.F., Mabrouki Y. (2020). The American Blue Crab *Callinectes sapidus* Rathbun, 1896 (Crustacea: Decapoda: Portunidae) is Rapidly Expanding Through the Mediterranean Coast of Morocco. Thalassas.

[4] Tagatz M.E. (1969). Some Relations of Temperature Acclimation and Salinity to Thermal Tolerance of the Blue Crab, *Callinectes sapidus*. Trans. Am. Fish. Soc..

[5] Hines A.H., Johnson E.G., Darnell M.Z., Rittschof D., Miller T.J., Bauer L.J., Rodgers P., Aguilar R., Kruse G.H., Eckert G.L., Foy R.J., Lipcius R.N., Sainte-Marie B., Stram D.L., Woodby D. (2010). Predicting effects of climate change on blue crabs in Chesapeake bay. Biology and Management of Exploited Crab Populations under Climate Change.

[6] Marchessaux G., Bosch-Belmar M., Cilenti L., Lago N., Mangano M.C., Marsiglia N., Sarà G. (2022). The invasive blue crab *Callinectes sapidus* thermal response: Predicting metabolic suitability maps under future warming Mediterranean scenarios. Front. Mar. Sci..

[7] Taylor D.L., Fehon M.M. (2021). Blue Crab (*Callinectes sapidus*) Population Structure in Southern New England Tidal Rivers: Patterns of Shallow-Water, Unvegetated Habitat Use and Quality. Estuaries Coasts.

[8] Mancinelli G., Chainho P., Cilenti L., Falco S., Kapiris K., Katselis G., Ribeiro F. (2017). On the Atlantic blue crab (*Callinectes sapidus* Rathbun 1896) in southern European coastal waters: Time to turn a threat into a resource?. Fish. Res..

[9] Marchessaux G., Mangano M.C., Bizzarri S., M’Rabet C., Principato E., Lago N., Veyssiere D., Garrido M., Scyphers S.B., Sarà G. (2023). Invasive blue crabs and small-scale fisheries in the Mediterranean sea: Local ecological knowledge, impacts and future management. Mar. Policy.

[10] Streftaris N., Zenetos A. (2006). Alien marine species in the Mediterranean—The 100 “worst invasives” and their impact. Mediterr. Mar. Sci..

[11] Kampouris T.E., Porter J.S., Sanderson W.G. (2019). *Callinectes sapidus* Rathbun, 1896 (Brachyura: Portunidae): An assessment on its diet and foraging behaviour, Thermaikos Gulf, NW Aegean Sea, Greece: Evidence for ecological and economic impacts. Crustac. Res..

[12] Nehring S. (2011). Invasion History and Success of the American Blue Crab *Callinectes sapidus* in European and Adjacent Waters. The Wrong Place—Alien Marine Crustaceans: Distribution, Biology and Impacts.

[13] Zotti M., De Pascali S.A., Del Coco L., Migoni D., Carrozzo L., Mancinelli G., Fanizzi F.P. (2016). H NMR metabolomic profiling of the blue crab (*Callinectes sapidus*) from the Adriatic Sea (SE Italy): A comparison with warty crab (*Eriphia verrucosa*), and edible crab (*Cancer pagurus*). Food Chem..

[14] Piras P., Esposito G., Meloni D. (2019). On the occurrence of the blue crab *Callinectes sapidus* (Rathbun, 1896) in Sardinian coastal habitats (Italy): A present threat or a future resource for the regional fishery sector?. Bioinvasions Rec..

[15] Khamassi F., Bahri W.R., Bhouri A.M., Chaffai A., Soufikechaou E., Ghanem R., Souissi J. (2022). Ben Biochemical composition, nutritional value, and socio-economic impacts of the invasive crab *Callinectes sapidus* Rathbun, 1896 in central Mediterranean Sea. Mediterr. Mar. Sci..

[16] Clavero M., Franch N., Bernardo-Madrid R., López V., Abelló P., Queral J.M., Mancinelli G. (2022). Severe, rapid and widespread impacts of an Atlantic blue crab invasion. Mar. Pollut. Bull..

[17] Nuñez M.A., Kuebbing S., Dimarco R.D., Simberloff D. (2012). Invasive Species: To eat or not to eat, that is the question. Conserv. Lett..

[18] NOAA (2023). Fisheries Economics of the United States 2020.

[19] Tarkan A.S., Tricarico E., Vilizzi L., Bİlge G., Ekmekçİ F.G., Filiz H., Giannetto D., İlhan A., Kİllİ N., Kirankaya G. (2021). Risk of invasiveness of non-native aquatic species in the eastern Mediterranean region under current and projected climate conditions. Eur. Zool. J..

[20] Martínez M.A., Velazquez G., Cando D., Núñez-Flores R., Borderías A.J., Moreno H.M. (2017). Effects of high pressure processing on protein fractions of blue crab (*Callinectes sapidus*) meat. Innov. Food Sci. Emerg. Technol..

[21] Nanda P.K., Das A.K., Dandapat P., Dhar P., Bandyopadhyay S., Dib A.L., Lorenzo J.M., Gagaoua M. (2021). Nutritional aspects, flavour profile and health benefits of crab meat based novel food products and valorisation of processing waste to wealth: A review. Trends Food Sci. Technol..

[22] Tufan B. (2023). Biochemical composition of different sex and body parts of blue crabs (*Callinectes sapidus*) caught from the middle Black Sea coast. Mar. Sci. Technol. Bull..

[23] Wu H., Ge M., Chen H., Jiang S., Lin L., Lu J. (2020). Comparison between the nutritional qualities of wild-caught and rice-field male Chinese mitten crabs (*Eriocheir sinensis*). LWT.

[24] Jiang X., Wang H., Cheng Y., Wu X. (2020). Growth performance, gonad development and nutritional composition of Chinese mitten crab *Eriocheir sinensis* selected for growth and different maturity time. Aquaculture.

[25] Ayas D. (2016). The Effects of Season and Sex on the Nutritional Quality of Muscle Types of Blue Crab *Callinectes sapidus* and Swimming Crab Portunus segnis. Nat. Eng. Sci..

[26] Küçükgülmez A., Çelik M., Yanar Y., Ersoy B., Çikrikçi M. (2006). Proximate composition and mineral contents of the blue crab (*Callinectes sapidus*) breast meat, claw meat and hepatopancreas. Int. J. Food Sci. Technol..

[27] FAO, WHO, UNU (1985). Energy and Protein Requirements. Report of a Joint FAO/WHO/UNU Expert Consultation.

[28] Gökoolu N., Yerlikaya P. (2003). Determinaton of proximate composition and mineral contents of blue crab (*Callinectes sapidus*) and swim crab (*Portunus pelagicus*) caught off the Gulf of Antalya. Food Chem..

[29] Çelik M., Türeli C., Çelik M., Yanar Y., Erdem Ü., Küçükgülmez A. (2004). Fatty acid composition of the blue crab (*Callinectes sapidus* Rathbun, 1896) in the north eastern Mediterranean. Food Chem..

[30] Alparslan Y., Metin C., Baygar T. (2022). Sex-and season-based comparison of lipid and fatty acid profiles of blue crab meat. J. Food Nutr. Res..

[31] Luvizotto-Santos R., Lee J.T., Branco Z.P., Bianchini A., Nery L.E.M. (2003). Lipids as Energy Source during Salinity Acclimation in the Euryhaline Crab *Chasmagnathus granulata* Dana, 1851 (Crustacea-grapsidae). J. Exp. Zool. Part A Comp. Exp. Biol..

[32] Kuley E., Özoǧul F., Özogul Y., Olgunoglu A.I. (2008). Comparison of fatty acid and proximate compositions of the body and claw of male and female blue crabs (*Callinectes sapidus*) from different regions of the Mediterranean coast. Int. J. Food Sci. Nutr..

[33] Martínez-Maldonado M.A., Velazquez G., Ramírez de León J.A., Borderías A.J., Moreno H.M. (2020). Effect of high pressure processing on heat-induced gelling capacity of blue crab (*Callinectes sapidus*) meat. Innov. Food Sci. Emerg. Technol..

[34] Pinheiro M.A.A., de Souza C.A., Borba H. (2015). Meat yield of the mangrove crab, *Ucides cordatus* (Linnaeus, 1763) (Crustacea, Brachyura, Ucididae). Bol. do Inst. Pesca.

[35] Galetti J.A., Calder B.L., Skonberg D.I. (2017). Mechanical Separation of Green Crab (*Carcinus maenas*) Meat and Consumer Acceptability of a Value-Added Food Product. J. Aquat. Food Prod. Technol..

[36] Galetti J.A. (2010). Mechanical Processing of European Green Crab (*Carcinus maenas*), the Development of a Value-Added Product and the Use of Restructuring Additives to Increase the Functional Properties of Green Crab Patties. Master’s Thesis.

[37] Gates K.W., Parker A.H. (1992). Characterization of Minced Meat Extracted from Blue Crab Picking Plant By-Products. J. Food Sci..

[38] Gates K.W., Parker A.H. (1993). Refrigerated and frozen storage characteristics of minced meat extracted from blue crab picking plant by-products. J. Aquat. Food Prod. Technol..

[39] Murphy M.G., Skonberg D.I., Camire M.E., Dougherty M.P., Bayer R.C., Briggs J.L. (2003). Chemical composition and physical properties of extruded snacks containing crab-processing by-product. J. Sci. Food Agric..

[40] Marchessaux G., Sibella B., Garrido M., Abbruzzo A., Sarà G. (2024). Can we control marine invasive alien species by eating them? The case of *Callinectes sapidus*. Ecol. Soc..

[41] Zotti M., Del Coco L., De Pascali S.A., Migoni D., Vizzini S., Mancinelli G., Fanizzi F.P. (2016). Comparative analysis of the proximate and elemental composition of the blue crab *Callinectes sapidus*, the warty crab *Eriphia verrucosa*, and the edible crab *Cancer pagurus*. Heliyon.

[42] Elsayed H.M., Hassanien F.R., Ibrahim S.M., Sulieman R.M. (2020). Biochemical Quality Indices of Blue Swimmer Crab (*Callinectes sapidus*) Meat. Adv. Anim. Vet. Sci..

[43] Zakzok S.M., Tawfik M.M., Mohammad S.H., Alkaradawe R.M. (2022). Biometric Study, Condition Factor and Biochemical Composition of the Blue Crab *Callinectes sapidus* Rathbun, 1896. J. Fish. Environ..

[44] Alparslan Y., Metin C., Emiroğlu S., Baygar T. (2023). Investigating the quality changes and shelf life of vacuum shrink-packaged raw and steam-cooked blue crabs under cold storage. Aquat. Res..

[45] Vidal J.L., Jin T., Lam E., Kerton F., Moores A. (2022). Blue is the new green: Valorization of crustacean waste. Curr. Res. Green Sustain. Chem..

[46] Su W., Yu S., Wu D., Xia M., Wen Z., Yao Z., Tang J., Wu W. (2019). A critical review of cast-off crab shell recycling from the perspective of functional and versatile biomaterials. Environ. Sci. Pollut. Res..

[47] Gao Y., Xu S., Yue Q., Wu Y., Gao B. (2016). Chemical preparation of crab shell-based activated carbon with superior adsorption performance for dye removal from wastewater. J. Taiwan Inst. Chem. Eng..

[48] Zapantioti A., Fotodimas I., Gkaliouri C., Ioannou Z. Valorization of blue crab (*Callinectes sapidus*) by-products as adsorbents. Proceedings of the 9th International Conference on Sustainable Solid Waste Management CORFU 2022.

[49] Maschmeyer T., Luque R., Selva M. (2020). Upgrading of marine (fish and crustaceans) biowaste for high added-value molecules and bio(nano)-materials. Chem. Soc. Rev..

[50] Venugopal V. (2022). Green processing of seafood waste biomass towards blue economy. Curr. Res. Environ. Sustain..

[51] Antunes-Valcareggi S.A., Ferreira S.R.S., Hense H. (2017). Enzymatic hydrolysis of blue crab (*Callinectes sapidus*) waste processing to obtain chitin, protein, and astaxanthin-enriched extract. Int. J. Environ. Agric. Res..

[52] Abd El-Ghany M.N., Hamdi S.A., Elbaz R.M., Aloufi A.S., El Sayed R.R., Ghonaim G.M., Farahat M.G. (2023). Development of a Microbial-Assisted Process for Enhanced Astaxanthin Recovery from Crab Exoskeleton Waste. Fermentation.

[53] Azelee N.I.W., Dahiya D., Ayothiraman S., Noor N.M., Rasid Z.I.A., Ramli A.N.M., Ravindran B., Iwuchukwu F.U., Selvasembian R. (2023). Sustainable valorization approaches on crustacean wastes for the extraction of chitin, bioactive compounds and their applications—A review. Int. J. Biol. Macromol..

[54] Pinheiro A.C.D.A.S., Martí-Quijal F.J., Barba F.J., Tappi S., Rocculi P. (2021). Innovative non-thermal technologies for recovery and valorization of value-added products from crustacean processing by-products—An opportunity for a circular economy approach. Foods.

[55] Ahmadkelayeh S., Hawboldt K. (2020). Extraction of lipids and astaxanthin from crustacean by-products: A review on supercritical CO_2_ extraction. Trends Food Sci. Technol..

[56] Sánchez-Camargo A.P., Meireles M.Â.A., Ferreira A.L.K., Saito E., Cabral F.A. (2012). Extraction of ω-3 fatty acids and astaxanthin from Brazilian redspotted shrimp waste using supercritical CO_2_+ ethanol mixtures. J. Supercrit. Fluids.

[57] Parthiban F., Balasundari S., Gopalakannan A., Rathnakumar K., Felix S. (2017). Comparison of the Quality of Chitin and Chitosan from Shrimp, Crab and Squilla Waste. Curr. World Environ..

[58] Rodrigues L.A., Matias A.A., Paiva A. (2021). Recovery of antioxidant protein hydrolysates from shellfish waste streams using subcritical water extraction. Food Bioprod. Process..

[59] Khiari Z., Kelloway S., Mason B. (2020). Turning Invasive Green Crab (*Carcinus maenas*) into Opportunity: Recovery of Chitin and Protein Isolate Through Isoelectric Solubilization/Precipitation. Waste Biomass Valoriz..

[60] Pires C., Marques A., Carvalho M.L., Batista I.B. (2017). Chemical Characterization of *Cancer pagurus*, Maja squinado, Necora puber and *Carcinus maenas* shells. Poultry Fish. Wildl. Sci..

[61] Mao X., Guo N., Sun J., Xue C. (2017). Comprehensive utilization of shrimp waste based on biotechnological methods: A review. J. Clean. Prod..

[62] Gildberg A., Stenberg E. (2001). A new process for advanced utilisation of shrimp waste. Process Biochem..

[63] Cahú T.B., Santos S.D., Mendes A., Córdula C.R., Chavante S.F., Carvalho L.B., Nader H.B., Bezerra R.S. (2012). Recovery of protein, chitin, carotenoids and glycosaminoglycans from Pacific white shrimp (*Litopenaeus vannamei*) processing waste. Process Biochem..

[64] Caruso G., Floris R., Serangeli C., Di Paola L. (2020). Fishery Wastes as a Yet Undiscovered Treasure from the Sea: Biomolecules Sources, Extraction Methods and Valorization. Mar. Drugs.

[65] Dey S.S., Dora K.C. (2014). Optimization of the production of shrimp waste protein hydrolysate using microbial proteases adopting response surface methodology. J. Food Sci. Technol..

[66] Gunasekaran J., Kannuchamy N., Kannaiyan S., Chakraborti R., Gudipati V. (2015). Protein Hydrolysates from Shrimp (*Metapenaeus dobsoni*) Head Waste: Optimization of Extraction Conditions by Response Surface Methodology. J. Aquat. Food Prod. Technol..

[67] Jiang W., Hu S., Li S., Liu Y. (2017). Biochemical and antioxidant properties of peptidic fraction generated from crab (*Portunus trituberculatus*) shells by enzymatic hydrolysis. Int. J. Food Sci. Technol..

[68] Kim S.B., Yoon N.Y., Shim K.B., Lim C.W. (2016). Antioxidant and angiotensin I-converting enzyme inhibitory activities of northern shrimp (*Pandalus borealis*) by-products hydrolysate by enzymatic hydrolysis. Fish. Aquat. Sci..

[69] Guerard F., Sumaya-Martinez M.T., Laroque D., Chabeaud A., Dufossé L. (2007). Optimization of free radical scavenging activity by response surface methodology in the hydrolysis of shrimp processing discards. Process Biochem..

[70] Yoon N.Y., Shim K.B., Lim C.W., Kim S.B. (2013). Antioxidant and angiotensin I converting enzyme inhibitory activities of red snow crab Chionoecetes japonicas shell hydrolysate by enzymatic hydrolysis. Fish. Aquat. Sci..

[71] Hajji S., Kchaou H., Bkhairia I., Ben Slama-Ben Salem R., Boufi S., Debeaufort F., Nasri M. (2021). Conception of active food packaging films based on crab chitosan and gelatin enriched with crustacean protein hydrolysates with improved functional and biological properties. Food Hydrocoll..

[72] Messina C.M., Manuguerra S., Arena R., Renda G., Ficano G., Randazzo M., Fricano S., Sadok S., Santulli A. (2021). In Vitro Bioactivity of Astaxanthin and Peptides from Hydrolisates of Shrimp (*Parapenaeus longirostris*) By-Products: From the Extraction Process to Biological Effect Evaluation, as Pilot Actions for the Strategy “From Waste to Profit”. Mar. Drugs.

[73] Syama Dayal J., Ponniah A., Imran Khan H., Madhu Badu P., Ambasankar K., Kumarguru Vasagam P. (2013). Shrimps—A nutritional perspective on JSTOR. Curr. Sci..

[74] Maia M.L., Grosso C., Barroso M.F., Silva A., Delerue-Matos C., Domingues V.F. (2023). Bioactive Compounds of Shrimp Shell Waste from Palaemon serratus and Palaemon varians from Portuguese Coast. Antioxidants.

[75] Hong S., Yuan Y., Yang Q., Zhu P., Lian H. (2018). Versatile acid base sustainable solvent for fast extraction of various molecular weight chitin from lobster shell. Carbohydr. Polym..

[76] Zou Y., Robbens J., Heyndrickx M., Debode J., Raes K. (2021). Bioprocessing of marine crustacean side-streams into bioactives: A review. J. Chem. Technol. Biotechnol..

[77] Ali M.H., Aljadaani S., Khan J., Sindi I., Aboras M., Aly M.M. (2020). Isolation and molecular identification of two chitinase producing bacteria from marine shrimp shell wastes. Pak. J. Biol. Sci..

[78] Cretton M., Malanga G., Mazzuca Sobczuk T., Mazzuca M. (2021). Lipid Fraction from Industrial Crustacean Waste and Its Potential as a Supplement for the Feed Industry: A Case Study in Argentine Patagonia. Waste Biomass Valoriz..

[79] Kaya M., Dudakli F., Asan-Ozusaglam M., Cakmak Y.S., Baran T., Mentes A., Erdogan S. (2016). Porous and nanofiber α-chitosan obtained from blue crab (*Callinectes sapidus*) tested for antimicrobial and antioxidant activities. LWT.

[80] Montoya J.M., Mata S.V., Acosta J.L., Cabrera B.E.H., Valdez L.G.L., Reyes C., Cureño H.J.B. (2021). Obtaining of astaxanthin from crab exosqueletons and shrimp head shells. Biointerface Res. Appl. Chem..

[81] Nekvapil F., Mihet M., Lazar G., Pinzaru S.C., Gavrilović A., Ciorîță A., Levei E., Tamaș T., Soran M.L. (2023). Comparative Analysis of Composition and Porosity of the Biogenic Powder Obtained from Wasted Crustacean Exoskeletonsafter Carotenoids Extraction for the Blue Bioeconomy. Water.

[82] Jabeen F., Younis T., Sidra S., Muneer B., Nasreen Z., Saleh F., Mumtaz S., Saeed R.F., Abbas A.S. (2021). Extraction of chitin from edible crab shells of *Callinectes sapidus* and comparison with market purchased chitin. Braz. J. Biol..

[83] Metin C., Alparslan Y., Baygar T., Baygar T. (2019). Physicochemical, Microstructural and Thermal Characterization of Chitosan from Blue Crab Shell Waste and Its Bioactivity Characteristics. J. Polym. Environ..

[84] Pinzaru S.C., Poplăcean I.-C., Maškarić K., Dumitru D.-A., Barbu-Tudoran L., Tămaș T.L., Nekvapil F., Neculai B. (2024). Raman Technology for Process Control: Waste Shells Demineralization to Produce Transparent Polymer Foils Reinforced with Natural Antioxidant, and Calcium Acetate By-Product. Processes.

[85] Baron R.D., Pérez L.L., Salcedo J.M., Córdoba L.P., Sobral P.J. (2017). do A. Production and characterization of films based on blends of chitosan from blue crab (*Callinectes sapidus*) waste and pectin from Orange (*Citrus sinensis* Osbeck) peel. Int. J. Biol. Macromol..

[86] Félix-Valenzuela L., Higuera-Ciapara I., Goycoolea-Valencia F., Argüelles-Monal W. (2001). Supercritical CO_2_/ethanol extraction of astaxanthin from blue crab (*Callinectes sapidus*) shell waste. J. Food Process Eng..

[87] Akbay İ.K., Güngör A., Özdemir T. (2022). Assessment of blue crab Shell (*Callinectes sapidus*) particles as bio-based filler to EPDM rubber. J. Appl. Polym. Sci..

[88] Nekvapil F., Ganea I.V., Ciorîță A., Hirian R., Ogresta L., Glamuzina B., Roba C., Pinzaru S.C. (2021). Wasted biomaterials from crustaceans as a compliant natural product regarding microbiological, antibacterial properties and heavy metal content for reuse in blue bioeconomy: A preliminary study. Materials.

[89] Mauricio-Sánchez R.A., Salazar R., Luna-Bárcenas J.G., Mendoza-Galván A. (2018). FTIR spectroscopy studies on the spontaneous neutralization of chitosan acetate films by moisture conditioning. Vib. Spectrosc..

[90] Ray L., Panda A.N., Mishra S.R., Pattanaik A.K., Adhya T.K., Suar M., Raina V. (2019). Purification and characterization of an extracellular thermo-alkali stable, metal tolerant chitinase from Streptomyces chilikensis RC1830 isolated from a brackish water lake sediment. Biotechnol. Rep..

[91] Xu T., Qi M., Liu H., Cao D., Xu C., Wang L., Qi B. (2020). Chitin degradation potential and whole-genome sequence of Streptomyces diastaticus strain CS1801. AMB Express.

[92] Asif T., Javed U., Zafar S.B., Ansari A., Ul Qader S.A., Aman A. (2020). Bioconversion of Colloidal Chitin Using Novel Chitinase from Glutamicibacter uratoxydans Exhibiting Anti-fungal Potential by Hydrolyzing Chitin Within Fungal Cell Wall. Waste Biomass Valoriz..

[93] Oyeleye A., Normi Y.M. (2018). Chitinase: Diversity, limitations, and trends in Engineering for suitable applications. Biosci. Rep..

[94] Aider M. (2010). Chitosan application for active bio-based films production and potential in the food industry: Review. LWT Food Sci. Technol..

[95] Ouattara B., Simard R.E., Piette G., Bégin A., Holley R.A. (2000). Diffusion of acetic and propionic acids from chitosan-based antimicrobial packaging films. J. Food Sci..

[96] Lorevice M.V., Otoni C.G., de Moura M.R., Mattoso L.H.C. (2016). Chitosan nanoparticles on the improvement of thermal, barrier, and mechanical properties of high- and low-methyl pectin films. Food Hydrocoll..

[97] Mahgoub S., Alagawany M., Nader M., Omar S.M., Abd El-Hack M.E., Swelum A., Elnesr S.S., Khafaga A.F., Taha A.E., Farag M.R. (2023). Recent Development in Bioactive Peptides from Plant and Animal Products and Their Impact on the Human Health. Food Rev. Int..

[98] Nekvapil F., Aluas M., Barbu-Tudoran L., Suciu M., Bortnic R.A., Glamuzina B., Pinzaru S.C. (2019). From Blue Bioeconomy toward Circular Economy through High-Sensitivity Analytical Research on Waste Blue Crab Shells. ACS Sustain. Chem. Eng..

[99] Aneesh P.A., Ajeeshkumar K.K., Lekshmi R.G.K., Anandan R., Ravishankar C.N., Mathew S. (2022). Bioactivities of astaxanthin from natural sources, augmenting its biomedical potential: A review. Trends Food Sci. Technol..

[100] Siahaan E.A., Agusman Pangestuti R., Shin K.H., Kim S.K. (2022). Potential Cosmetic Active Ingredients Derived from Marine By-Products. Mar. Drugs.

